# Evaluation of Risk and Preventive Factors for Necrotizing Enterocolitis in Premature Newborns. A Systematic Review of the Literature

**DOI:** 10.3389/fped.2022.874976

**Published:** 2022-05-17

**Authors:** Ana M. Campos-Martinez, J. Expósito-Herrera, M. Gonzalez-Bolívar, E. Fernández-Marin, Jose Uberos

**Affiliations:** ^1^Neonatal Intensive Care Unit, San Cecilio Clinical Hospital, Granada, Spain; ^2^Pediatrics Department, Medicine Faculty, Granada, Spain

**Keywords:** necrotizing enterocolitis, enteral nutrition, human milk, dysbiosis, probiotics

## Abstract

Necrotizing enterocolitis (NEC) is a serious condition related to prematurity and the initiation of enteral feeding. In this article, we review the evidence published in recent years on necrotizing enterocolitis risk factors (prematurity, feeding with low-weight formula, existence of intestinal dysbiosis) and protective factors (human milk or donated milk supply, supplementation of human milk with oligosaccharides, probiotics administration, and the determination of disease predictive biomarkers). A systematic review was conducted of preventive, risk and predictive factors for necrotizing enterocolitis in neonates prior to 37 weeks' gestational age, based on a literature search for clinical trials, meta-analyses, randomized controlled trials and systematic reviews published between January 2018 and October 2021. For this purpose, the PubMed, MEDLINE, and Cochrane Library databases were consulted. The literature search obtained 113 articles, of which 19 were selected for further analysis after applying the inclusion and exclusion criteria. The conclusions drawn from this analysis were that adequate knowledge of risk factors that can be prevented or modified (such as alteration of the intestinal microbiota, oxidative stress, metabolic dysfunction at birth, or alteration of the immunity modulation) can reduce the incidence of NEC in premature infants. These factors include the supplementation of enteral nutrition with human milk oligosaccharides (with prebiotic and immunomodulatory effects), the combined administration of probiotics (especially the *Lactobacillus* spp and *Bifidobacterium* spp combination, which inhibits bacterial adhesion effects, improves the intestinal mucosa barrier function, strengthens the innate and adaptive immune system and increases the secretion of bioactive metabolites), the supplementation of human milk with lactoferrin and the use of donated milk fortified in accordance with the characteristics of the premature newborn. The determination of factors that can predict the existence of NEC, such as fecal calprotectin, increased TLR4 activity, and IL6 receptor, can lead to an early diagnosis of NEC. Although further studies should be conducted to determine the values of predictive biomarkers of NEC, and/or the recommended doses and strains of probiotics, lactoferrin or oligosaccharides, the knowledge acquired in recent years is encouraging.

## Introduction

In premature infants, necrotizing enterocolitis (NEC) is the leading cause of death due to gastrointestinal disease, affecting 5–12% of very low birth weight (VLBW) newborns (1, 2).

The pathophysiology of necrotizing enterocolitis is characterized by its multifactorial nature. Among the most common risk factors are those of prenatal origin, such as chorioamnionitis or genetic imprinting; perinatal factors such as low gestational age at birth, low birth weight, or abnormal gut microbiota (3); and risk factors derived from neonatal care and stage, such as mechanical ventilation, type of feeding, or pharmacological interventions (1).

Various strategies have been proposed for the prevention of NEC (4), including the routine use of different strains of probiotics, especially *Lactobacillus* and *Bifidobacterium* (5). However, the most suitable combination of strains and the optimum doses have yet to be determined.

In the newborn, microbiota are mainly formed by species of *Bifidobacterium*. The colonization of the newborn in the first stages of life is influenced by several factors, including the type of delivery, the type of infant feeding, gestational age at birth and the administration of antibiotics in the early stages of life. In cesarean deliveries, the intestinal colonization of the newborn may be delayed, coinciding with neonatal colonization with flora from the maternal skin. In vaginal deliveries, on the other hand, colonization occurs preferentially with flora from the birth canal. After cesarean delivery, newborns present a lower proportion of *Bacteroides* with fewer *Bifidobacteria*. Recent studies have reported a decrease in *Bifidobacteria* in the fecal flora of the newborn when the mother received intrapartum prophylaxis with ampicillin; however, other bacteria of the genus *firmicutes*, to which *Lactobacillus* and *Clostridia* belong, are not modified (6).

Human milk is another factor preventing NEC, both because of the microbiota it provides and because of the presence of immunoglobulin A and oligosaccharides (4). During lactation, the bacteria associated with the intestine are transported by the blood and lymphatics to the mammary gland. Human milk promotes a flora rich in *Bifidobacterium*, with lower counts of *E. coli, Clostridium difficile*, and *Bacteroides fragilis*. Formula milk is known to alter the profile of intestinal colonization, associating it with more *Bifidobacterium* and other facultative anaerobes.

After childbirth, breastfeeding contributes to increasing the initial inoculum with lactic acid-producing bacteria, *Bifidobacterium*, and bacteria from the mother's skin. *Bifidobacterium* and fecal *Lactobacillus/Enterococci* counts at 6 months are higher in breastfed infants than in formula-fed infants (7). Skin-to-skin contact, the administration of lactoferrin to human milk, the use of donated milk with fortification individualized to the characteristics of the premature newborn (8), as well as the determination of factors that can predict the existence of NEC, such as fecal calprotectin, increased TLR4 activity and IL6 receptor (9), can all modify the NEC outcome.

The aim of this study is to review the existing evidence on preventive and risk factors for necrotizing enterocolitis in premature infants.

## Materials and Methods

This study is a systematic review. The review was conducted of preventive, risk and predictive factors for necrotizing enterocolitis in neonates prior to 37 weeks' gestational age, based on a literature search for clinical trials, meta-analyses, randomized controlled trials, and systematic reviews published between January 2018 and October 2021. This review was performed via a search of the following websites presenting data on relevant clinical practice: the Cochrane Library, PubMed and Medline databases. In PubMed, the MeSH terms used were “Necrotizing enterocolitis”[Mesh] AND “Infant, Very Low Birth Weight”[Mesh] OR “Necrotizing enterocolitis”[Mesh] AND “Probiotics”[Mesh] OR “Necrotizing enterocolitis”[Mesh] AND “Nutrition”[Mesh] OR “Necrotizing enterocolitis”[Mesh] AND “Preventive factor”[Mesh] OR “Necrotizing enterocolitis”[Mesh] AND “Risk factor”[Mesh] AND 2018[PDAT]: 2021[PDAT] AND (English[lang] OR Spanish[lang]) AND (Clinical Trial[ptyp] OR Meta-Analysis [ptyp] OR Practice Guideline [ptyp] OR Randomized Controlled Trial [ptyp] OR Review [ptyp]).

We use the PRISMA 2020 chek list for this systematic review (10).

The following selection criteria were applied: (a) Premature infants with <37 weeks' gestational age or <2,500 g birth weight; (b) Studies published during the period 2018 to 2021; (c) Studies focused on risk or preventive factors from NEC (primary or secondary outcomes); (d) Comparison between intervention groups, with placebo or negative control.

The exclusion criteria were: (a) Articles in languages other than Spanish or English; (b) Studies dealing exclusively with animals; (c) Articles that presented insufficient data or confusing data; (d) Articles that did not differentiate between premature infants and other age groups.

We assessed the risk of bias and extracted the following data: general study information (author's name, publication year), study population details, details of the intervention and comparison and outcomes.

Of the articles used, 13 had the existence or not of necrotizing enterocolitis as the primary outcome of their study, but in any case, even if the outcome was secondary, the presented significant statistical data regarding the NEC development or not.

For each study, we look at and assess the odds ratio (OR) or relative risk (RR), and 95% confidence intervals (IC) on the results.

**Figure 1:**
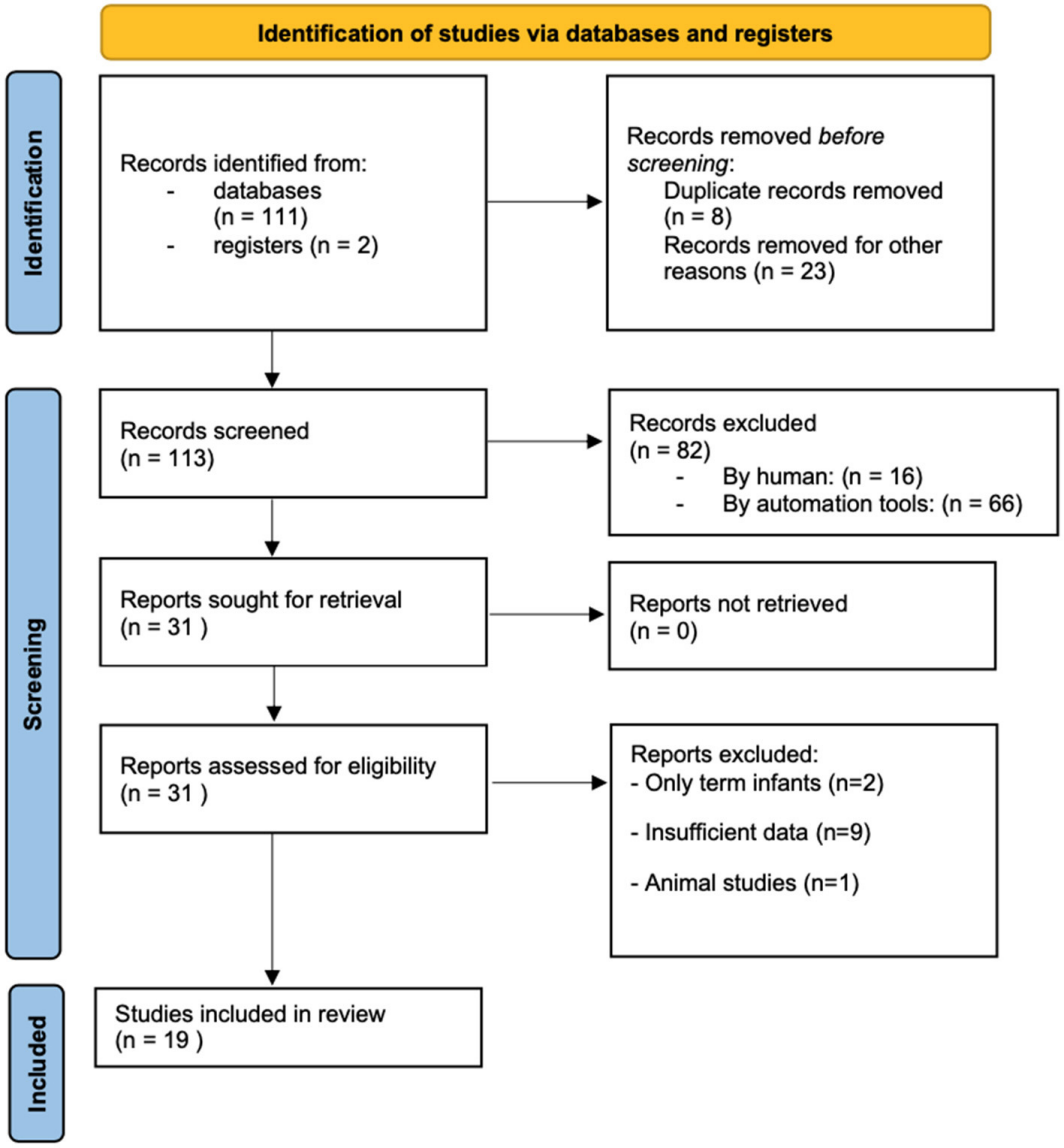
PRISMA 2020 flow diagram of the studies selected for analysis.

The PRISMA 2020 flow diagram for the source selection process is shown in Figure 1.

## Results

The literature search initially yielded 113 articles. After discarding duplicates and excluding unrelated articles (according to the document title and abstract), 31 papers remained, for which the full texts were obtained. Following application of the inclusion/exclusion criteria described, eleven of these papers were excluded, leaving nineteen for the final analysis (Figure 1).

The following studies were included in the final analysis: seven systematic reviews (SR), eight meta-analyses (NMA), two narrative reviews (NR), one cohort study (OS), and one case-control study (CC).

Table 1 summarizes the main results obtained, showing the type of study, sample size (it it can be specified) and the factors studied: preventive factors (probiotics, oligosaccharides, human and donated milk, lactoferrin, inmunomoulators, and other factors), risk factors and predictive factors for NEC.

**Table 1:** Characteristics of the studies included in the systematic review.

**Study**	**Characteristics of patients**	**Sample size**	**OR (95% CI)**	**Intervention**	**Results**
**NEC preventive factors**					
**1. Probiotics**					
Robertson et al. (3) (OS) NEC is the primary outcome	<36 w GA + <1,500 g	*n*: 982	0.44 (0.23–0.85)	*Lactobacillus + Bifidobacterium*	The incidence of NEC was reduced with the administration of *Lactobacillus* and *Bifidobacterium* compared to those who did not receive it.
Beghetti et al. (5) (NMA) NEC is the primary outcome	<37 w GA	*n*: 10,664	0.32 (0.00–0.21)	*Lactobacillus* *Bifidobacterium* Formula feed vs. breastmilk	*Lactobacillus acidophilus* LB had an effect in reducing NEC in both groups, taking breast milk or artificial milk *Bifidobacterium lactis* Bb12/b94 was associated with decreased NEC in stage >2, especially in the breast milk subgroup.
Patel and Underwood (11) (NMA) NEC is the primary outcome	<34 w GA	*n*: 10,520	0.58 (0.37–0.91)	*Lactobacillus o Bifidobacterium*	The combination of *Lactobacillus acidophilus* and *Bifidobacterium bifidum* implies a lower risk of NEC suggestive of surgical intervention Other combinations of probiotics reduce the risk of NEC, requiring more studies to determine the combination and dose.
Baldassarre et al. (12) (NMA) NEC is NOT the primary outcome	<34 w GA or <1,500 g	n: not stated	Not defined	*Lactobacillus + Bifidobacterium*	The use of probiotics (*Lactobacillus* + *Bifidobacerium*) reduces the incidence of NEC in premature infants with <34 weeks' gestational age or <1,500 g birth weight.
Liu et al. (13) (NMA) NEC is NOT the primary outcome	<37 w GA	*n*: 11,045	0.36 (0.24–0.53)	*Lactobacillus rhamnosus* GG *Lactobacillus reuteri* Others	The use of different probiotics in premature patients significantly reduces the risk of NEC compared to untreated patients The use of *L. rhamnosus* GG and *L. reuteri* in doses lower than 10^9^ CFU is postulated to be more beneficial, due to the risk of bacterial translocation.
Morgan et al. (14) (NMA) NEC is NOT the primary outcome	<37 w GA <2,500 g	*n*: 15,712	0.56 (0.39–0.80)	*Lactobacillus + Bifidobacterium* *Bacillus + Enterococcus* *Bifidocbacterium* + *Streptoccus* isolate	They reviewed 63 clinical trials of probiotic supplementation vs. placebo treatment, finding that the combination of one or more strains of *Lactobacillus* spp and *Bifidobacterium* spp reduced all-cause mortality included NEC.
Underwood (15) (NMA) NEC is the primary outcome	<37 w GA <28 w GA >1,000 g	*n*: 85,596 *n*: 4,683	*p* <0.0002 *p* <0.005	*Lactobacillus + Bifidobacterium*	Use of probiotics reduces mortality and NEC incidence in all premature infants, especially in <28 GA and <1,000 g birth weight.
**2. Oligosaccharides (HMOS), breast milk, donated milk**					
Nolan et al. (16) (NR) NEC is the primary outcome	<1,000 g <37 w GA	*n*: 291 *n*: 27,749	0.84 (0.79–0.88)	HMOs breastmilk	Higher breast milk concentrations of HMO disialyllacto-N-tetraose (DSLNT) is associated with a lower risk of NEC (Bell stage 2 and 3 combined).
Altobelli et al. (17) (NMA and OS) NEC is the primary outcome	<37 w GA	*n*: ~10,484	0.62 (0.42–0.93)	Breast/donated vs. formula milk	Reduced risk of NEC>2 in preterm infants who received breast or donated milk instead of formula (even when the amount of breast or donated milk was 50%).
Miller et al. (18) (NMA and SR) NEC is NOT the primary outcome	<28 w GA ≤ 1,500 g	*n*: 16,422	Not defined in OR ARR 4.3–3.8%	Breast vs. formula milk	Maternal milk reduces NEC.
Quigley et al. (19) (SR) NEC is NOT the primary outcome	<37 w GA	*n*: 1,809	1.87 (1.23–2.85)	Breast vs. donated milk	Feeding with formula milk compared with donor human milk, results in higher rates of weight gain, linear growth, and head growth and a higher risk of developing NEC.
Sánchez-Luna et al. (8) (NR) NEC is NOT the primary outcome	<37 w GA	*n*: 659	Not defined in OR Reduction of NEC from 10.9 to 2.4%	Personalized nutrition with pasteurized donated milk + /– fortifier	Personalized nutrition with donated milk decreases the risk of NEC.
**3. Lactoferrin**					
Pammi and Suresh (20) (SR and OS) NEC is the primary outcome	<37 w GA	*n*: 5,370	1.10 (0.86–1.41)	Supplementation with lactoferrin	Decreases late-onset sepsis (both suspected and confirmed, and confirmed only) but not NEC ≥ stage II.
**4. Inmunomodulators**					
Nolan et al. (21) (SR) NEC is the primary outcome	<37 w GA	Not specified	Not defined	Immunomodulatory components of breast milk	The dietary intake of the breastfeeding mother has been shown to influence the variability of human milk concentrations of fat-soluble and water-soluble vitamins and other nutrients. These nutrients, including immunoglobulins, growth factors, cytokines, and immune cells, have been demonstrated to transfer from the mother to the neonate through breast milk.
Hackam et al. (22) (SR) NEC is the primary outcome	<37 w GA	Not specified	Not defined	Role of TLR4 in the development of NEC.	Breast milk is a powerful TLR4 inhibitor, while mutations in TLR4 genes lead to increased NEC risk in humans, providing proof-of-concept for its role in NEC.
Alganabi et al. (23) (SR) NEC is the primary outcome	<1,500 g	Not specified	Not defined	TLR4 Factors related to NEC Breast milk and HMOs in the prevention of NEC	There are factors involved in the pathophysiology of NEC, such as nitric oxide, toll-like receptor 4 (TLR4), microvascular blood flow that produces intestinal ischemia, and reduced activity of stem cells at the intestinal level Breast milk and the supplementation of enteral nutrition with oligosaccharides from breast milk (HMO) cause a greater functioning of stem cells at the intestinal level, significantly reducing the risk of NEC.
**5. Other preventive factors**					
Rose and Patel (24) (SR) NEC is the primary outcome	<37 w GA <37 w GA <2,000 gr <37 w GA <1,500 g <32 w GA	*n*: 4,702 *n*: 1,170 *n*: 2,869 *n*: 750 *n*: 285 *n*:1,092	0.50 (0.32–0.78) 0.30 (0.10–0.89) 0.43 (0.21–0.87) Any stage of NEC:0.38 (0.23–0.64) Death due to NEC: 0.18 (0.03–1) 0.93 (0.64–1.34) 1.07 (0.67–1.70)	Prenatal steroids Use of progesterone in the mother in preterm labor Hydric restriction Oral or parenteral supplementation with arginine Delay in the start of enteral nutrition Trophic enteral nutrition	The use of antenatal steroids reduces the risk of NEC by 50%, but in <25 w GA it does not present benefits in this aspect Progesterone given to women at risk for preterm birth decreased the relative risk of NEC Total fluid goals for premature infants are influenced by multiple factors but a restricted approach may decrease NEC risk Meta-analysis of small trials demostrate that oral or parenteral supplementation with arginine decreases the risk for any stage of NEC and death due to NEC Delay in enteral feeding by <1,500 g or <32 w GA does not alter the risk of NEC, including children with RIC Trophic enteral nutrition does not modify the risk of NEC.
**NEC risk factors**					
Rose and Patel 2018 (24) (SR) NEC is the primary outcome	≤ 31 w GA <37 w GA <37 w GA	*n*: 4,649 *n*: 5,003 *n*: 874	2.09 (1.30–2.35) 1.25 (0.12–12.50) 0.44 (0.17–0.12)	Reduced placental flow at delivery Antibiotics administration Anemia	Reduced placental flow during preterm delivery increases the risk of NEC. A recent meta-analysis of observational studies found prolonged antibiotic exposure was associated with NEC, but no statistically significant effect on NEC when looking at whether or not an infant received prophylactic antibiotics or broad vs. narrow spectrum antibiotics. Anemia ( ≤ 8 g/dl) was associated with an increased risk for NEC but not red cell transfusion or erythropoiesis-stimulating agents used to modify transfusion.
**NEC predictive factors**					
Sinclair et al. (25) (CC) NEC is the primary outcome	<32 w GA	*n*: 995	*p* <0.001	Metabolite levels (aminoacids and acylcarnitine) at birth and in the days of life 1, 7, 28, 42	Analytical leves of of alanine, phenylalanine, free carnitine, C16, arginine, C14:1/C16, and citrulline/phenylalanine in day 1 were associated with later development of NEC. Over time, differences in individual levels associated with NEC onset changed from predominantly AA at birth to predominantly AC at day 42. Subjects who developed NEC received significantly lower weight-adjusted total calories.
Nguyen and Sangild (9) (SR) NEC is the primary outcome	<37 w GA	Not specified	Not defined	Study of biomarkers (calprotectin, IL6, IL17) for NEC prediction	NEC gut tissues also display increased activation of TLR4 signalingand formation of neutrophil extracellular traps (NETs) with release of antimicrobial neutrophil components, including calprotectin. As a result, fecal calprotectin has been suggested as a potential biomarker for NEC diagnosis. The polarization of CCR9^+^ Treg into CCR9^+^ IL17 producing Treg was regulated by IL6 activity. The authors therefore proposed that IL6 receptor antibody can be used to treat NEC. The exciting results from this study may pave the way for future investigations on new disease blood biomarkers as well as therapeutic approaches with inhibition of gut chemokines or IL-6 signaling.

*OS, observational study; NMA, network meta-analysis; NR, narrative review; MA, meta-analysis; SR, systematic review; CC, cases-controls; OR, odds ratio*.

### NEC Preventive Factors

#### Probiotics

About the relationship of probiotics with the reduction in the risk of NEC, we selected 7 studies (1 OS, 6 NMA), with ~134.000 patients, of <37 weeks (the sample size can be consulted in Table 1), showing that the administration of different strains of *Lactobacillus and Bifidobacterium* (3, 5, 11–15), compared with the use of the other probiotics or placebo, reduces the incidence of NEC Bell stage ≥2, especially in <28 AG and <1,000 g birth weight (15). In one of the studies included, belonging to Patel and Underwood (11), it is assessed that the combination of *Lactobacillus acidophuilus* and *Bifidobacterium bifidum* in <34 weeks, implies a decrease both in NEC and in presenting NEC that requires surgical intervention. Other authors such as Liu et al. (13), propose *Lactobacillus rhamnosus GG* and *Lactobacillus reuteri* at doses <10^9^ CFU as the best combination of probiotics in <37 weeks, due to the risk of bacterial translocations.

In four of the seven articles included in this section, the appearance or not of NEC was part of their primary outcomes. Other outcomes studied were the reduction in mortality and the appearance or not of late sepsis. In both cases, the incidence was lower in the probiotics administration group (*Lactobacillus and Bifidobacterium*).

#### Oligosaccharides, Human Milk, Donated Milk

Five studies (8, 16–19) were collected in this section to assess whether the use of oligosaccharides from breast milk, and the administration of donated and human milk vs. formula, reduced the incidence of NEC. Of the 5 studies (2 NR, 1 SR, 2 NMA), 2 of them presented the development or not of NEC as the primary outcome. Nolan et al. (16) reviewed a series of studies with a sample size of 27.749 preterms <37 weeks (291 patients <1,000 g), to determine whether human milk oligosaccharides (HMOs) contributed to the prevention of NEC. The results obtained suggest that a higher human milk concentration of HMO disialyllacto-N-tetraose (DSLNT) is associated with a lower risk of the infant developing NEC (Bell stages 2 and 3 combined).

Three studies (17–19) with a sample size of 28.715 patients, show the benefit of using breast milk and donated milk instead of formula milk in reducing the risk of developing NEC Bell stage ≥2, even showing benefit in this reduction when mixed feeding is taken instead of only formula milk (19).

#### Lactoferrin

Pammi and Suresh (20) conducted a systematic review of the preventive role of lactoferrin in NEC and reported low-certainty evidence from studies of good methodological quality that lactoferrin supplementation of enteral feeds decreases late-onset sepsis (both suspected and confirmed, and confirmed only) but not NEC ≥ stage II.

#### Inmunomodulators

This section contains 3 systematic reviews in which the results on the role that immunomodulators in breast milk have on the development of NEC have been evaluated.

In the first study in this section, led by Nolan et al. (21), observed that major immunologic components in human milk, such as secretory immunoglobulin A (IgA), and growh factors, protecting against NEC.

The other 2 sistematic reviews (22, 23), showed that NEC develops in response to exaggerated bacterial signaling in the premature intestine, as a consequence of elevated expression and activity of the bacterial receptor toll-like receptor 4 (TLR4), which is important for normal gut development. Human milk is a powerful TLR4 inhibitor, while mutations in TLR4 genes lead to increased NEC risk in humans, providing proof-of-concept for its role in NEC.

#### Other Preventive Factors

Rose and Patel (24) performed an systematic review with an extensive critical analysis of the preventive factors. With respect to maternal and neonatal factors, these authors observed that antenatal steroid (ANS) administration decreased the relative risk of NEC by 50%, in preterm infants, but they reported no benefit with respect to NEC from the use of ANS at very low gestational ages, perhaps due to effects on the competing cause of death (more survivors increase the number of infants at risk for NEC). Progesterone given to women at risk for preterm birth decreases the relative risk of NEC by 70%. Total fluid goals for premature infants are influenced by multiple factors, but a restricted approach may decrease the risk of NEC in infants <2,000 g (*n* = 526 infants).

On the other hand, oral or parenteral supplementation with arginine (26) decreases the risk for any stage of NEC and death due to NEC. Delayed feeding in infants <1,500 g or <32 weeks does not alter the NEC risk even for IUGR infants with abnormal umbilical Doppler flow velocities. Therefore, the best feeding strategy for the most immature infants remains unclear. A systematic review (27) included in the Rose and Patel work, showed that the question of trophic feeding vs. enteral fasting in extremely low birth weight infants has yet to be clarified. In patients <1500 g suggests there is no increased risk of NEC with trophic feeding.

### NEC Risk Factors

Within this section, we value various studies collected in the systematic review prepared by Rose and Patel (24), observing the negative impact that some factors have on the appearance of NEC. Among factors related to intrauterine growth, compromised fetal blood flow before or at the time of delivery may result in fetal ischemia, contributing to NEC (20).

### NEC Predictive Factors

Two studies were included in this section (1 SR and 1 CC). In both studies, the primary outcome was the NEC development or not. Sinclair et al. (25) published a multicenter case-control study with 995 patients <32 weeks, and hypothesized that in addition to nutritional variability, metabolic dysfunction is associated with the onset of NEC, specially the measure of amino acid (AA) and acylcarnitine (AC) in blood. A review by Nguyen and Sangild (9) reported a significant reduction in lamina propria Treg density in the ileum of NEC infants, suggesting impaired intestinal Treg function and excessive inflammatory responses during NEC progression. As a result, fecal calprotectin has been suggested as a potential biomarker for NEC diagnosis.

## Discussion

Necrotizing enterocolitis is a serious health problem for premature newborns. Knowledge of the protective, risk, and predictive factors of this disease will help us reduce its incidence.

### NEC Preventive Factors

Preventive factors against the development of NEC are becoming clearer. These include breastfeeding or donated human milk, the combined use of probiotics from different strains of *Lactobacillus* and *Bifidobacterium*, the exogenous administration of oligosaccharides from human milk (which are a protective factor for the immature intestine), and other immunomodulators, especially from human milk. On the other hand, controversy remains about the benefit of the systematic administration of lactoferrin.

Different studies have shown the preventive effect of the administration of different strains of *Lactobacillus* + *Bifidobacterium* on the development of NEC in premature infants <37 weeks (3, 5, 11, 12, 14, 15). In the works by Patel and Underwood (11), and Baldasarre et al. (12), the patients included in the study are <34 weeks, but one of the limitations in the study by Baldasarre et al. (12) is that it does not specify the sample size analyzed. Underwood (15) analyzed patients <28 weeks and <1,000 g, with similar results to patients of higher gestational age. In the work of Beguetti et al. (5), they studied the effect of different probiotics on NEC, differentiating infants given formula milk from those who were breastfed. These authors reported that the administration of *Lactobacillus acidophilus* reduced the risk of NEC (OR 0.03; 95% CI 0.00–0.21) in both groups, while the use of *Bifidobacterium lactis* Bb12/b94 was associated with fewer cases of NEC in stage >2. Liu et al. (13) found that the relative risk (RR) ratio of developing NEC in premature infants given probiotics was 0.33 (95% C.I 0.24–0.46), indicating a powerful preventive effect. A limitation of this study was that different probiotics were used and in different doses. *Lactobacillus rhamnosus* GG and *Lactobacillus reuteri* are among the best candidates. However, caution should be taken not to exceed doses of 10^9^ CFU in premature neonates because of the risk of bacterial translocation.

Nolan (16) reviewed evidence on the association between the presence of oligosaccharides in human milk and the incidence of NEC, observing that this incidence decreased with HMO supplementation. Similarly, Alganabi et al. (23) highlighted the role played by HMOs in enhancing the functioning of stem cells at the intestinal level, thereby reducing the risk of NEC.

In the last study of this section, Sanchez Luna et al. (8) conducted a narrative review. They established a personalized nutrition unit (PNU), providing donated milk and fortification appropriate to individual need. After the introduction of this PNU, mother's-own-milk (MOM) feeding increased from 90% of the premature infants to 98.8%, and exclusive MOM feeding increased from 39 to 55%. The authors also reported a clear difference in total protein content by gestational age and lactation, by the existence of the PNU was also associated with decreased NEC, although it is true that the existence or not of necrotizing enterocolitis was not the primary outcome of this study.

About the inmunomodulators of human milk, Nolan et al. (21) reviewed the immutable components of human milk and their protective influence on necrotizing enterocolitis. Major immunologic components in human milk, such as secretory immunoglobulin A (IgA) and growth factors, are known to play a role in regulating gut barrier integrity and microbial colonization, there by protecting against NEC. Human milk supplements infants with human milk oligosaccharides, leukocytes, cytokines, nitric oxide, and growth factors, all of which attenuate inflammatory responses and provide immunological defenses to reduce the incidence of NEC, and both Hackham et al. (22) and Alganabi et al. (23) examined the role of human milk as an inhibitor of TLR4. Given that certain mutations in the TLR4 genes increase the risk of NEC, this effect of human milk would protect against enterocolitis in such cases.

One of the limitations of the Rose and Patel (24) study, was that they performed a systematic review with an extensive critical analysis of the preventive factors, and some studies are old, so they have been discarded. Some of the preventive factors analyzed in this study, and that they present validity for their study, are the use of prenatal steroids (especially in infants older than 25 w), the use of EPO or red blood cell transfusions, fluid restriction, and probiotic supplementation. Delay in enteral feeding in newborns weighing <1,500 g or with a gestational age of <32 w does not alter the risk of NEC, even for infants with intrauterine growth restriction. Neither is this risk modified by trophic enteral nutrition.

### NEC Risk Factors

Known risk factors for NEC include prenatal factors (genetics, chorioamnionitis, intestinal immaturity), perinatal factors (low gestational age, low birth weight, abnormal colonization of the intestinal microbiota) and those derived from the neonatal stage and the care provided during this phase (environmental stress, mechanical ventilation, central catheters, pharmacological interventions, or antibiotic therapy) (1). In a cohort of infants aged <34 weeks (28), those with a persistent ductus arteriosus (PDA) with or without indomethacin treatment had an increased risk of NEC compared to infants with no PDA.

Among other factors that may favor the appearance of NEC find reduced placental flow, fluid overload, anemia (Hb ≤ 8 g/dl, but not red cell transfusion) or the prolonged use of antibiotics (24).

### NEC Predictive Factors

Sinclair et al. (25) considered the levels of metabolites (amino acids and acylcarnitine) at birth and on days 1, 7, 28, and 42 of life. This analysis revealed higher levels of acylcarnitine and lower levels of amino acids in infants with NEC, and so these factors should be taken into account as possible predictors of NEC. Finally, Nguyen and Sangild (9) studied various predictive biomarkers of NEC, including fecal calprotectin and IL 6 and 17: this review reported a significant reduction in lamina propria Treg density in the ileum of NEC infants, suggesting impaired intestinal Treg function and excessive inflammatory responses during NEC progression. Furthermore, NEC gut tissues also display increased activation of TLR4 signaling and the formation of neutrophil extracellular traps (NETs) with the release of antimicrobial neutrophil components, including calprotectin. As a result, fecal calprotectin has been suggested as a potential biomarker for NEC diagnosis.

Although some of these studies were only performed in the laboratory or used small samples, they provide a valuable basis for further research that may achieve more reliable and consistent results.

## Conclusions

The number of studies conducted to determine the risk and preventive factors for NEC is constantly increasing. In this respect, the use of probiotics, especially *Lactobacillus* and *Bifidobacterium* in combination, is becoming widespread as a preventive measure, although the best strains and optimum doses have yet to be established. However, supplementation with oligosaccharides from human milk is known to be beneficial, and there is no doubt that both human milk and donated milk have a protective effect against NEC, due to the presence of specific microbiota, HMOs, metabolites, and immunomodulatory factors. The likelihood of NEC can be reduced by avoiding formula milk and the prolonged use of antibiotics and by preventing neonatal anemia. A better understanding of new biomarkers, such as IL 6 or IL 17, would facilitate the early identification of NEC and thus reduce its incidence.

## Data Availability Statement

The original contributions presented in the study are included in the article/supplementary material, further inquiries can be directed to the corresponding author/s.

## Author Contributions

All authors listed have made a substantial, direct, and intellectual contribution to the work and approved it for publication.

## Conflict of Interest

The authors declare that the research was conducted in the absence of any commercial or financial relationships that could be construed as a potential conflict of interest.

## Publisher's Note

All claims expressed in this article are solely those of the authors and do not necessarily represent those of their affiliated organizations, or those of the publisher, the editors and the reviewers. Any product that may be evaluated in this article, or claim that may be made by its manufacturer, is not guaranteed or endorsed by the publisher.
